# A Landscape Approach to Invasive Species Management

**DOI:** 10.1371/journal.pone.0160417

**Published:** 2016-07-29

**Authors:** Miguel Lurgi, Konstans Wells, Malcolm Kennedy, Susan Campbell, Damien A. Fordham

**Affiliations:** 1 The Environment Institute and School of Biological Sciences, The University of Adelaide, Adelaide, SA 5005, Australia; 2 Environmental Futures Research Institute, Griffith University, Brisbane, QLD 4111, Australia; 3 Department of Agriculture and Food, Western Australia, 3 Baron-Hay Ct, South Perth, WA 6151, Australia; 4 Department of Agriculture and Food, Western Australia, 444 Albany Hwy, Albany, WA 6330, Australia; Deakin University, Australia, AUSTRALIA

## Abstract

Biological invasions are not only a major threat to biodiversity, they also have major impacts on local economies and agricultural production systems. Once established, the connection of local populations into metapopulation networks facilitates dispersal at landscape scales, generating spatial dynamics that can impact the outcome of pest-management actions. Much planning goes into landscape-scale invasive species management. However, effective management requires knowledge on the interplay between metapopulation network topology and management actions. We address this knowledge gap using simulation models to explore the effectiveness of two common management strategies, applied across different extents and according to different rules for selecting target localities in metapopulations with different network topologies. These management actions are: (i) general population reduction, and (ii) reduction of an obligate resource. The reduction of an obligate resource was generally more efficient than population reduction for depleting populations at landscape scales. However, the way in which local populations are selected for management is important when the topology of the metapopulation is heterogeneous in terms of the distribution of connections among local populations. We tested these broad findings using real-world scenarios of European rabbits (*Oryctolagus cuniculus*) infesting agricultural landscapes in Western Australia. Although management strategies targeting central populations were more effective in simulated heterogeneous metapopulation structures, no difference was observed in real-world metapopulation structures that are highly homogeneous. In large metapopulations with high proximity and connectivity of neighbouring populations, different spatial management strategies yield similar outcomes. Directly considering spatial attributes in pest-management actions will be most important for metapopulation networks with heterogeneously distributed links. Our modelling framework provides a simple approach for identifying the best possible management strategy for invasive species based on metapopulation structure and control capacity. This information can be used by managers trying to devise efficient landscape-oriented management strategies for invasive species and can also generate insights for conservation purposes.

## Introduction

Biological invasions continue to be a major threat to biodiversity worldwide [1], having strong detrimental effects on ecological communities, local economies and production systems [2–3]. Once established, invasive species often form collections of local populations that can be linked at regional scales. The structure of this connected set of populations is often determined by the interplay between the behaviour of dispersing individuals, structural elements of the landscape and environmental processes [4–7]. Therefore the control and eradication of invasive species is a landscape-scale problem, often making local management strategies inadequate for the control, or eradication of established invasive species. Historically, the control of terrestrial invasive species has been largely based on general population reduction by trapping, shooting or poisoning in focal areas of the landscape [8]. Even though active population reduction has been effective at decreasing wildlife populations, its effectiveness is very much dependent on the life-history traits of the focal species [9].

Landscape-scale approaches to wildlife management have long been adopted in marine and terrestrial conservation biology [10–13], motivated by the loss of wildlife populations in degraded and fragmented landscapes and seascapes [14]. Landscape-scale control has been actively promoted as best practice management for a number of established invasive species (e.g. [15–17]); however, these management actions have often failed to consider the distribution and connectivity of local populations across the landscape. This is despite modelling frameworks now being available to forecast the spread of invasive species in spatial settings, which explicitly account for metapopulation structure (e.g., [18]). Demographic traits are also important for determining the success of an invasive species [19–20], and should be incorporated into studies of invasive species management. For example, Cassey et al. [20] recently used a non-spatial modelling approach to identify demographic traits associated with invasiveness and to better direct effective management intervention. Where demographic and genetic traits and metapopulation dynamics have been incorporated into models of invasive species, these approaches have generally used an overly simplistic spatial population structure (e.g. assuming that all local populations are equally connected) [16–17, 21]. Explicitly considering metapopulation dynamics (i.e. the structure of the network of local populations) and demographic processes (e.g., dispersal, intrinsic rate of population growth, density dependence, etc.) when managing invasive species will enhance the effectiveness of invasive species control at the landscape scale [21], with potential added benefits for the conservation of native biodiversity and agricultural production [15].

To meet this knowledge gap we developed a population network model grounded in metapopulation theory [22] and used the model to provide a stronger understanding of how management strategies for invasive species are sensitive to landscape configuration. Our novel modelling approach links the effects of network-based spatial management interventions with metapopulation structure to improve assessments of the efficacy of alternative strategies for managing invasive species. Spatial configurations of local populations are combined with varying local management intensities and regional management extents, to produce a broad range of potential landscape-scale invasive species management scenarios. The efficacy of these management scenarios − spatial arrangements of treatments − is then tested for different spatial population structures using *in-silico* experiments on model and real world metapopulation networks. We assess our modelling approach using two different management actions, reducing resource availability and targeted removal of individuals. Both of these management actions have been extensively used, independently and in tandem, for the management of invasive European rabbits (*Oryctolagus cuniculus*) in Australia and their on-ground effects have been assessed [23]. For example, warren ripping is considered an efficient mechanical control of rabbits in some regions of Australia because it directly kills animals and reduces access to an obligate resource (i.e. warrens) [24–26]. Additionally, in many areas of Australia, poison baiting is used to control rabbit population abundance [23], whereby baiting on farmland depends on the commitment of individual farmers. It remains largely unknown whether different metapopulation network structures require different spatially explicit baiting strategies.

Rabbits are mobile animals with high reproductive rates [27]. They are among the worst pest species in Australia [28–29]. Because of their high intrinsic rate of population increase [30], we hypothesise that management actions based on the mid- to long-term reduction of an obligate resource, which is not easily restored, will have stronger effects on metapopulation persistence than general population reduction. Additionally, since dispersal of individuals through highly connected networks of populations is likely to counteract efficient management by quick re-colonisation and population recovery, we hypothesise that management results should be most apparent at moderate to high levels of control.

We use our novel model-based framework to evaluate the potential effects of different management strategies on the control of the European rabbit across 10 landscapes in Western Australia. This region was chosen because of its value as a biodiversity hotspot and area of conservation concern [31–32]. These two features make this region an important focus of conservation and pest-management efforts. Even though the specific landscapes chosen are primarily agricultural in nature, it is expected that the effects of controlling rabbits in these areas, which are embedded within a region of high biodiversity, will nonetheless yield conservation benefits. Rabbit control is thus of interest for both conservation and production objectives in the studied area.

We identify important demographic and spatial processes of rabbits and underlying landscapes that should be considered when developing spatial management strategies. Our results provide recommendations that are relevant not only for the management of rabbits in Australia but also for the management of invasive species more generally–especially for species where targeting resource availability has been identified as an efficient management strategy (e.g. nesting sites for house crows in Singapore [33], or for monk parakeets in the US [34]).

## Methods

We developed a demographic-based metapopulation model that allows the efficacy of different management strategies and levels of effort for controlling invasive species to be tested at regional scales. Local population dynamics are modelled using a logistic-type population growth function. Spatial dynamics are governed by dispersal processes occurring between local populations arranged in a given landscape configuration. System-level metapopulation dynamics (i.e., changes in overall abundances at the regional scale) thus emerge from the interaction between these two processes. We allowed management strategies to vary not only in the level in which local populations are managed but also in the extent (the number of local populations managed in a metapopulation) and the spatial arrangement of management efforts. In doing so, we consider different topologies of both metapopulations and management efforts, allowing us to test how these two processes interact and affect control effectiveness.

### Local population dynamics and dispersal

Population dynamics at the local scale were modelled using a Ricker logistic growth equation, a mathematical equation commonly used to model species’ population growth [35]. Thus, changes in local abundance (*N*) through time are governed by Eq (1):

$$N_{t+1}=N_{t}\;*\;e^{r*(1-\frac{N_{t}}{K})+\varepsilon }$$ (1)

where *t* is the current time step of the model, *r* is the intrinsic growth rate of population growth for the species, and *K* is its carrying capacity. Stochasticity is incorporated into the demographic model through the term ε, which is added to the populations’ growth rate (Eq (1)). ε is drawn from a zero-mean normal distribution with and standard deviation σ = 0.31 (see below).

We derived parameter estimates for the model by fitting Eq (1) to long-term rabbit abundance data for a population in Australia (S1 Table) [36]. Parameter values were estimated using the PATS library for R [37], which yielded the following values: *r* = 0.43, *K* = 160, and σ = 0.31. Populations going below a threshold of 10 individuals were considered to go extinct and abundance set to 0 based on [38]. This allows for recovery of local populations when they are above 10 individuals at a single time step, either through immigration or population growth or a combination of the two [21].

Dispersal processes between local populations allowed for the incorporation of regional dynamics into the metapopulation networks. Rabbit dispersal was governed by a negative exponential function that reaches values of less than half around 5 km away from the centre of the population [39]. We used the following dispersal (*dk*) function:

$$dk=e^{-\phi *dist}$$ (2)

where *ϕ* was set to 1/5 to incorporate the information that the majority of dispersal events will occur within 5 km of the population; and *dist* is the particular distance for which the *dk* probability of dispersal is to be obtained. This function allows for rare, long-distance, dispersal events. Aside from the probability of dispersal to a given distance *dk*, dispersal in the model is also affected by the fraction of individuals dispersing (*d*). We employed 5 different values of dispersal in our simulations, i.e. *d* < 0, 0.1, 0.3, 0.6, 0.9 (zero corresponds to isolated populations).

### Metapopulations from model networks

**Fig 1:**
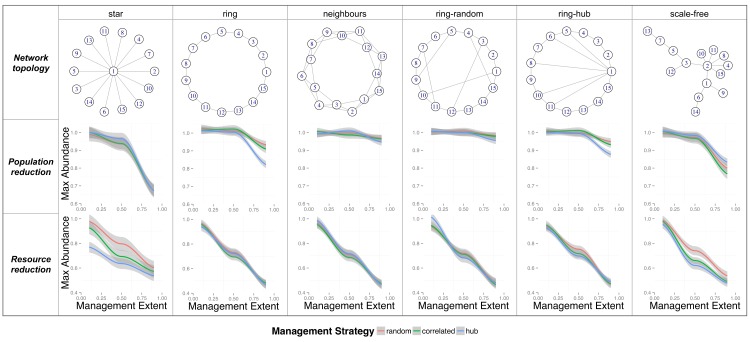
Changes in maximum metapopulation abundance in response to different management strategies and the underlying structure of model metapopulation networks. Top row shows the topology of model metapopulation networks. Middle and bottom rows show the effects of applying the management actions of general population reduction and reduction of resource availability, respectively, over the corresponding metapopulation pictured in the top row. Effects are measured as the relative change in maximum metapopulation abundance after vs. before management is applied. Dispersal (*d*) = 0.3 and management level (*l*) = 0.6. Management extent (*e*) varies between 0.1 and 0.9. Lines are a local polynomial regression fit to 100 replicates for each value of *e* and shadows represent the standard error of the mean. Colours represent different spatial management strategies (*s*): red = *random*, green = *correlated*, blue = *hub*.

To generate metapopulations from model networks we used six different model network configurations (each composed of 15 local populations), based on commonly studied network structures with well-defined structural and dynamical properties [40], and which varied in the way links between local populations are arranged. The particular network topologies we have explored are: *star*, *ring*, *neighbours*, *ring-random*, *ring-hub*, and *scale-free*, which are defined in Fig 1 (see ‘*network topology*’ row). Metapopulation topologies obtained from model networks thus ranged in complexity from homogeneously (e.g., ring-like model networks) to heterogeneously (e.g., star model networks) connected populations, allowing us to directly investigate the importance of metapopulation topology on invasive species’ management.

### Rabbit metapopulations in real-world landscapes

**Fig 2:**
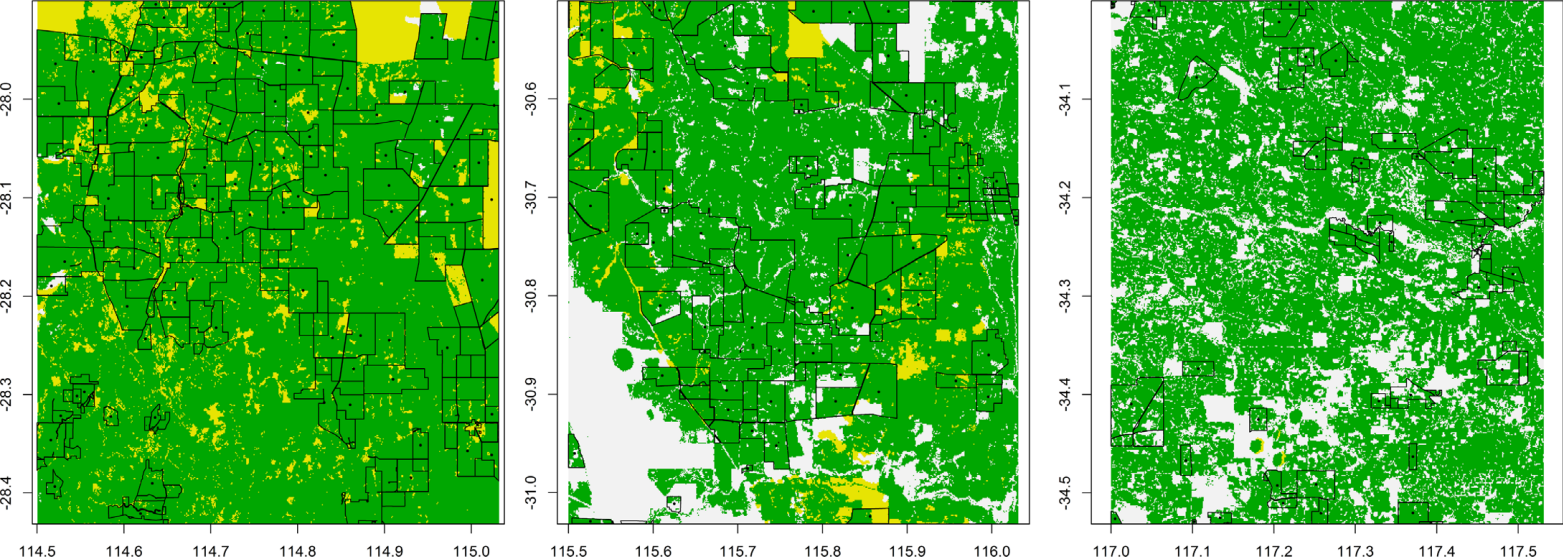
Examples of landscapes for rabbit populations in Western Australia. Shown randomly selected real-world landscapes of 50km^2^ extent from which networks of local population connectivity were obtained. Green represents grassland and arable land (suitable as foraging habitat by rabbits); yellow represents shrubland and open woodlands (suitable as shelter habitat by rabbits); and white shows habitat not suitable for forage or shelter (see text). Land cover classification is based on the Australian National Vegetation Information System database (NVIS) at 100*100m resolution. Black lines represent managed farmland units and dots the geometric centres of these units. Farmland units are typically considered in local and regional rabbit control practices.

We generated metapopulations based on real-word landscapes in ten randomly selected 50km^2^ regions of southwestern Western Australia (latitude -27.5 to -35, longitude 114.3 to 119.3; see Fig 2 for examples of the landscapes employed). The majority of native vegetation in the focal areas has been cleared for agriculture, providing relatively homogeneous habitat for rabbits and other species. We categorised vegetation types on 100*100m resolution from the Australian National Vegetation Information System database (NVIS), version 4.1 (Australian Department of the Environment, accessible at: http://www.environment.gov.au/land/native-vegetation/national-vegetation-information-system). We used expert advice to reclassify vegetation types: patches covered by grassland and arable land (cleared, non-native vegetation) were classed as *forage habitat*; shrubland and open woodlands as *shelter habitat*; and all other vegetation types as *not suitable habitat* for foraging or shelter, but suitable for dispersal. Some relatively small urban vegetation patches (or of other vegetation types able to support rabbits), which are known to harbour rabbits, were not captured in the vegetation surveys and hence our modelled landscapes. However, these habitats were rare and would not have biased our results.

Rabbits can only survive if both sufficient forage and shelter habitat is available [41]. We assumed that each hectare of: (i) forage habitat can provide resources for five rabbits, based on estimates from semiarid grasslands in Australia [42]; and (ii) shelter habitat can harbour a maximum of 100 rabbits based on average values from detailed studies of population densities in natural populations in Western Australia (our study region) [43], where they report rabbit densities between 30 and 150 per ha.

To convert these real-world landscapes into rabbit metapopulations, we calculated the number of rabbits in each 5 km^2^ cell (100 cells per landscape) based on the number of fine-resolution cells (100*100m) covered with forage and shelter habitats in each coarse cell. We defined connections between populations from different cells as the Euclidean distance between the central coordinates of cells. Only cell distances < 20 km were considered as connected, as rabbits are unlikely to disperse over larger distances [39]. We considered natal dispersal in our models, but not home range movement; and assume that a fraction of the new individuals from each local rabbit population can move to neighbouring populations at each modelled time step based on a dispersal kernel (see above). Although our conversion of information on landscape composition and configuration (i.e. vegetation maps) into rabbit metapopulations is likely to be a relatively coarse approximation, we nevertheless expect our approach to capture the overall structure of how rabbits are distributed in Western Australia reasonably well. Although other factors such as predators and/or biological control activity might change the metapopulation structure dynamically, we consider vegetation as the main driver of metapopulation structure. This simplifies the model design while still ensuring a realistic metapopulation structure.

### Management scenarios

We compared the effectiveness of two management actions: (1) general population reduction (through for example, poison baiting, which is common management practice in Western Australia [43]), and (2) reduction of an obligate resource and its subsequent effect on the carrying capacity of local populations. For rabbits, the reduction of an obligate resource may refer to the elimination of an essential resource at the local population scale such as the destruction of shelter and/or nesting sites. Although resource reduction of this kind would be more difficult to achieve in Western Australia, since rabbits do not typically build warrens for shelter, it is common practice in other areas of Australia [24–26]. Furthermore, removal of nesting sites has been identified as a feasible and effective control strategy for other invasive species (e.g. [33–34]). Resource reduction in our model reduces the population as soon as it is applied, simulating the effects of warren ripping in the wild in Australia. We tested the relative impact of population reduction versus resource reduction on metapopulation dynamics as well as the effectiveness of different pest-management applications for rabbits.

For each of the two management actions described above we performed combinatorial *in-silico* experiments that involved the variation of three further aspects of management: (1) management extent (*e*) (i.e., the proportion of local populations being managed); *e* ∈ {0.1, 0.5, 0.9} (‘∈‘ indicates a value for *e* is an element of the three values) (2) management level (*l*) at the local scale, which refers to either the fraction of the population being removed or the proportional reduction in carrying capacity, depending on whether the control action being simulated is general population reduction or resource reduction, respectively; *l* ∈ {0.3, 0.6, 0.9}, and (3) spatial management strategy (*s*) of local populations.

The spatial management strategies (*s*) employed took three distinct forms: (i) local populations to be managed were randomly chosen across the landscape (*random*); (ii) the first local population to be managed is chosen randomly and then its direct neighbours in the metapopulation are chosen until the total number of populations meets the value of *e* (*correlated*); (iii) the most connected population is chosen first and subsequently its neighbours (*hub*). In the *correlated* scenario, if all the neighbour populations have been chosen and the number of populations to be managed has not been reached, another (not managed) population is chosen randomly and the process is repeated. If all the neighbour populations have been chosen and the number of populations to be managed has not been reached for the *hub* scenario, the next most connected is selected. The *random* strategy represents management efforts without any planning because it does not involve any landscape-scale information. In contrast the *hub* strategy, exemplifies the other end of the continuum, in which knowledge of the regional connectivity of the landscape and associated metapopulation structure is used to inform the coordination of management efforts.

### Model simulations

The combination of different values for the various aspects of management described above resulted in 55 management scenarios (including a *null* non-management scenario). In combination with the four different values for dispersal *d*, this resulted in 220 different scenarios. For each of these we simulated 100 replicates consisting of 90 simulated time steps of Eq (1) for each local population, where each time step represents one year in population growth. This resulted in a total of 220,000 simulations. Because of the stochastic nature of the population model, different outcomes are possible among replicates.

Initial population abundances were taken from a normal distribution centred on the carrying capacity of local populations (*K* = 160) and standard deviation equal to 10% of the carrying capacity (σ = 16). Management was applied after a transient (i.e. burn-in) period of 70 time steps and for a total of 10 time steps, in the case of general population reduction. Management was applied for one time step in the case of reduction in carrying capacity, and carrying capacity remained in the reduced state for the rest of the simulation. This simulates situations where coordinated, ongoing, management actions results in sustained (or ongoing) removal of a critical resource. In the case of resource removal through warren ripping, we assume that resource removal is continually adopted in a coordinated manner to achieve and sustain low rabbit populations [25], although in situations with less intensive management, rabbits can potentially re-open ripped warrens. We then let the simulations run a further 10 time steps and compared maximum metapopulation size and its variance before and after the application of the respective management action (i.e., before vs. after management).

Source code for the software implementation of the model is available in S1 Appendix.

### Statistical analyses

To evaluate the effectiveness of different management strategies, we employed three different summary statistics: (i) proportional change in maximum abundance of the metapopulation, a measure of the relative decrease in maximum metapopulation abundance, (ii) proportional change (i.e. the ratio) in the coefficient of variation (CV) of maximum metapopulation abundance, a measure typically used to assess population stability [44], and (iii) fraction of surviving populations after management, a measure of the persistence of local populations in the landscape. Mean abundance was also considered as a summary statistic and the results obtained were similar to those for maximum abundance.

We used boosted regression trees (BRTs) [45] with learning rate of 0.0001, a bag fraction of 0.5, and a tree complexity of 5, to analyse the relative importance of the effects of *e* (management extent), *l* (management level), *d* (dispersal) and *s* (spatial management strategy) on each of the three model outputs. Additionally, local polynomial regression fitting was applied to identify trends in the response variables. Linear models (LMs) were used to test the effect of management strategy (*s*) on relative decrease in maximum abundance–all other parameters being fixed. All simulations and statistical analyses were performed in R [46]. Local polynomial regression fitting was done using the *loess* function. BRTs were done using the *gbm*.*step* function from the *dismo* package in R.

## Results

The degree to which management strategies differed in their impact on decreasing maximum metapopulation abundance was mainly driven by management extent (*e*). Additionally, our simulations showed that resource reduction is likely to provide a more efficient method for managing invasive species (such as rabbits in Australia) when compared to general population reduction (Fig 1).

### Managing invasive species through general population reduction in model networks

The largest effect of general population reduction on model metapopulation networks was ~ 30% decrease in maximum metapopulation abundance over 10 time steps for intermediate levels of dispersal (*d* = 0.3) and management level (*l* = 0.6). There were no noticeable differences in the effect among the three spatial management strategies (*s*) (Fig 1). When dispersal was low (i.e., *d* = 0 or *d* = 0.1) the effectiveness of removing individuals increased (S1 Fig).

The effectiveness of management efforts generally improved in more heterogeneous model networks in comparison to homogeneous ones, regardless of the spatial management strategy employed (Fig 1). Pronounced changes in maximum abundance were only observable in *star* and *scale-free* networks, where metapopulation abundance decreased by 30 and 20%, respectively, for large management extents (*e* = 0.9). In other more homogeneous model networks (e.g., *ring*, *neighbours*), maximum metapopulation abundance decreases were much smaller (5 to < 20%).

The *hub* spatial management strategy was more efficient in decreasing maximum metapopulation abundance than the other two (*random* and *correlated*) when applied to ring-like networks such as *ring* and *ring-hub* (particularly for large management extents, *e* = 0.9). However, the strength of this effect was reduced as populations become more connected to others, as for example, in the case of the *neighbours* network (see middle row in Fig 1). This result shows that for metapopulations where local patches are arranged in a ring-like configuration, only being able to exchange individuals with one neighbour patch to either side, management of neighbouring populations is the most efficient option (Fig 1).

When dispersal is high (e.g., *d* = 0.6) and management level is large (e.g., *l* = 0.9—resulting in 90% decrease in metapopulation abundance), managing randomly selected populations is a better strategy then *hub* and *correlated* in homogeneous landscapes (e.g., ring-type networks; S2 Fig). This is true however, only when management extent (*e*) is high (i.e., a high fraction of local populations is managed).

Our *in-silico* experiments on model metapopulation networks, therefore, show that when the management aim is a general reduction of populations, spatial strategies targeting more central populations in the network, such as the *hub* strategy, are more efficient for the control of invasive species metapopulations; at least in the narrow circumstances of ring-like arrangement of populations. Additionally, heterogeneous networks of local populations such as those presenting *star* or *scale-free* topologies are more amenable to management when large extents are achieved (*e* = 0.9).

### Managing invasive species by reducing an obligate resource in model networks

Reducing carrying capacity (*K*) by 60% (i.e., *l* = 0.6) in 90% of local populations (i.e., *e* = 0.9) resulted in decreases in metapopulation abundance of up to 50% regardless of the management strategy for intermediate levels of dispersal (*d* = 0.3) (Fig 1). In contrast, implementing a similar reduction in *K* in 10% of the populations (*e* = 0.1) resulted in only ~ 10% decrease in metapopulation abundance. A synergistic effect involving demographic stochasticity and dispersal is responsible for this difference, as larger decreases in *K* result in larger fluctuations around the populations’ carrying capacity. Excess individuals are then sent out to neighbouring populations, increasing in this way the ability of the metapopulation to maintain larger numbers. Different spatial management strategies (*random*, *correlated*, *hub*) showed similar results for low and high values of *e* when a reduction in *K* was applied, but differed at intermediate levels of *e*.

When managing 50% of the local populations (i.e., *e* = 0.5), significant differences arose between spatial management strategies but only for particular model networks. When local populations were arranged in a *star* network, it was more efficient to employ *hub* or *correlated* spatial management strategies than a *random* one (local management) (Fig 1 and Table 1). Similarly, *scale-free* landscape arrangements are likely to be managed more efficiently by employing regional concerted efforts (*hub* or *correlated*) than local (*random*) strategies (Fig 1 and Table 1).

**Table 1:** Effects of spatial management strategies (*s*) on the relative decrease in maximum metapopulation abundance in model networks when an obligate resource (K) is reduced in 50% of the local populations.

Network topology	Spatial management	Coefficient	Std. error	P-value
*star*	*correlated*	-0.10	0.03	< 0.01
	*hub*	-0.16	0.03	< 0.001
*scale-free*	*correlated*	-0.08	0.02	< 0.01
	*hub*	-0.12	0.02	< 0.001
*ring-hub*	*correlated*	-0.04	0.03	0.18
	*hub*	-0.06	0.03	< 0.05
*ring*	*correlated*	-0.04	0.02	0.14
	*hub*	-0.01	0.02	0.72
*ring-random*	*correlated*	-0.01	0.02	0.7
	*hub*	-0.04	0.02	0.11
*neighbours*	*correlated*	-0.02	0.02	0.3
	*hub*	0.01	0.02	0.72

LMs results describe the difference between the spatial management strategies (correlated and hub) and a null (*random*) spatial strategy. Dispersal (*d*) = 0.3, management level (*l*) = 0.6. The sign of the coefficient value represents the directionality of the relationship between the spatial management strategy and the relative decrease in metapopulation abundance.

No significant differences were found among different spatial management strategies applied to the other (more homogeneous) model metapopulation networks (Fig 1 and Table 1). Only a slight improvement in management was found in *ring-hub* type networks when applying a *hub* management strategy vs. a *random* one (Table 1). Although *ring-hub* metapopulations have highly connected patch networks, the fact that the other populations have a similar number of neighbours counteracts any potential effect of using a hub strategy to manage the more central patch/patches. This is also true for the other homogeneous networks (*ring*, *neighbours*, and *ring-random*).

### Managing rabbits at landscape scales in Western Australia

**Fig 3:**
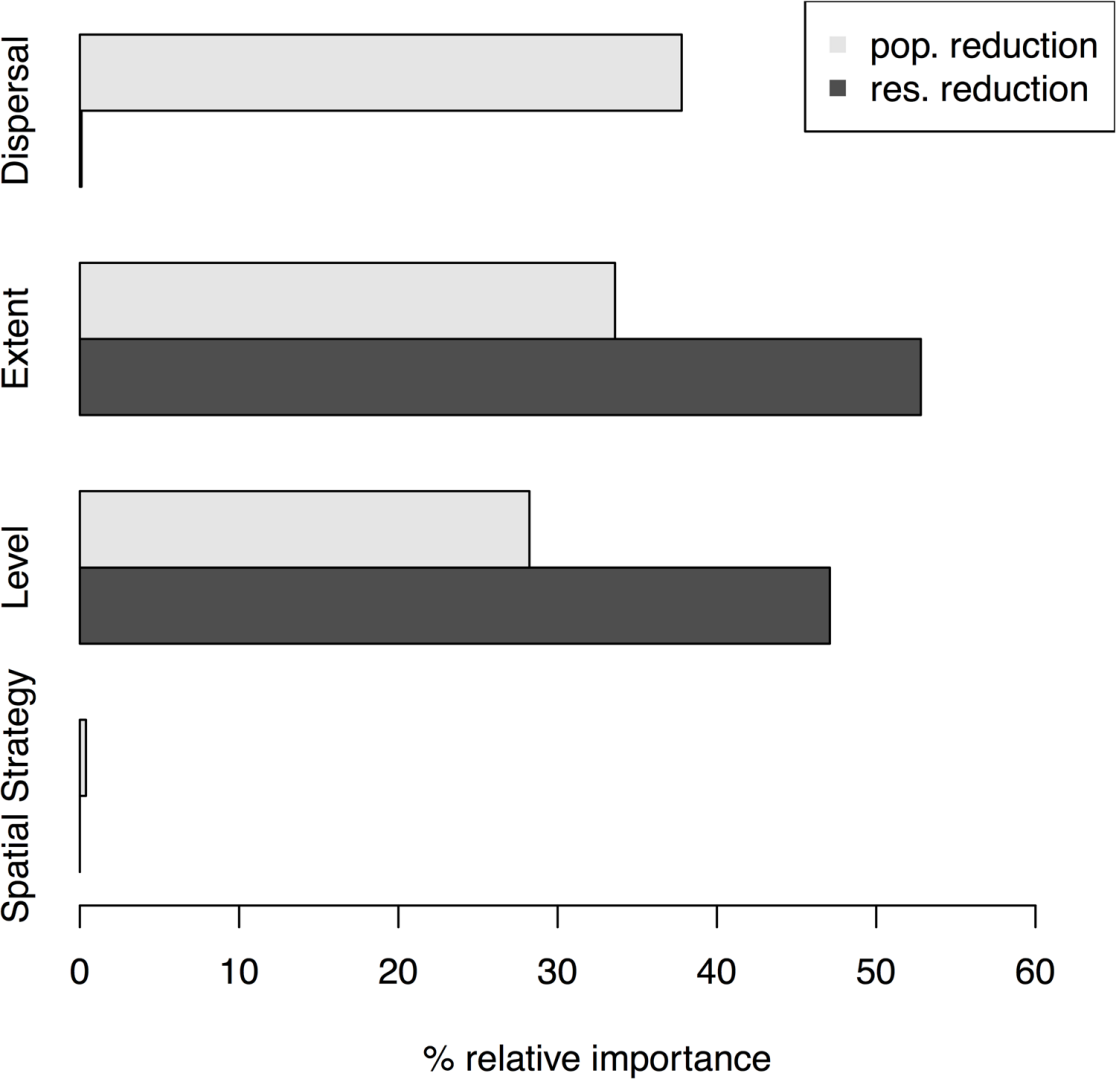
Relative importance of various components of management and dispersal on the change in maximum abundance of managed rabbit metapopulations. Partial effects plots showing the relative importance of dispersal, management extent, management level, and spatial management strategy on maximum rabbit abundance at the metapopulation scale. Management actions are: population reduction (pop. reduction; light grey), and reduction of an obligate resource (res. reduction; dark grey).

Partial effects plots from boosted regression trees showed that dispersal (relative importance (*ri*) = 38%), management extent (*ri* = 33%) and management level (*ri* = 28%) had the strongest effects on decreases in metapopulation abundance of rabbits when individuals were removed from the real-world landscapes (Fig 3). When management was focused on reducing an obligate resource, the most important factors determining the effect of different management scenarios on decrease in metapopulation abundance were management extent (*ri* = 53%) and management level (*ri* = 47%) (Fig 3). Notably, applying different spatial management strategies in very homogenous real-world landscapes had negligible effects on metapopulation abundance for both types of management (Fig 3).

The fraction of surviving populations was affected in the same way when general population reduction was performed: dispersal, management extent, and level, in that order, were the most influential factors determining local population persistence (S3 Fig). In contrast, when reducing an obligate resource, management level became the most influential factor, followed by dispersal, extent, and spatial strategy (S3 Fig). BRT ranking of the different components of management was similar for the effect of both management actions on metapopulation stability: dispersal (*ri* = 40–50%), management level (*ri* = 25–30%), and extent (*ri* = 25–30%) (S4 Fig).

**Fig 4:**
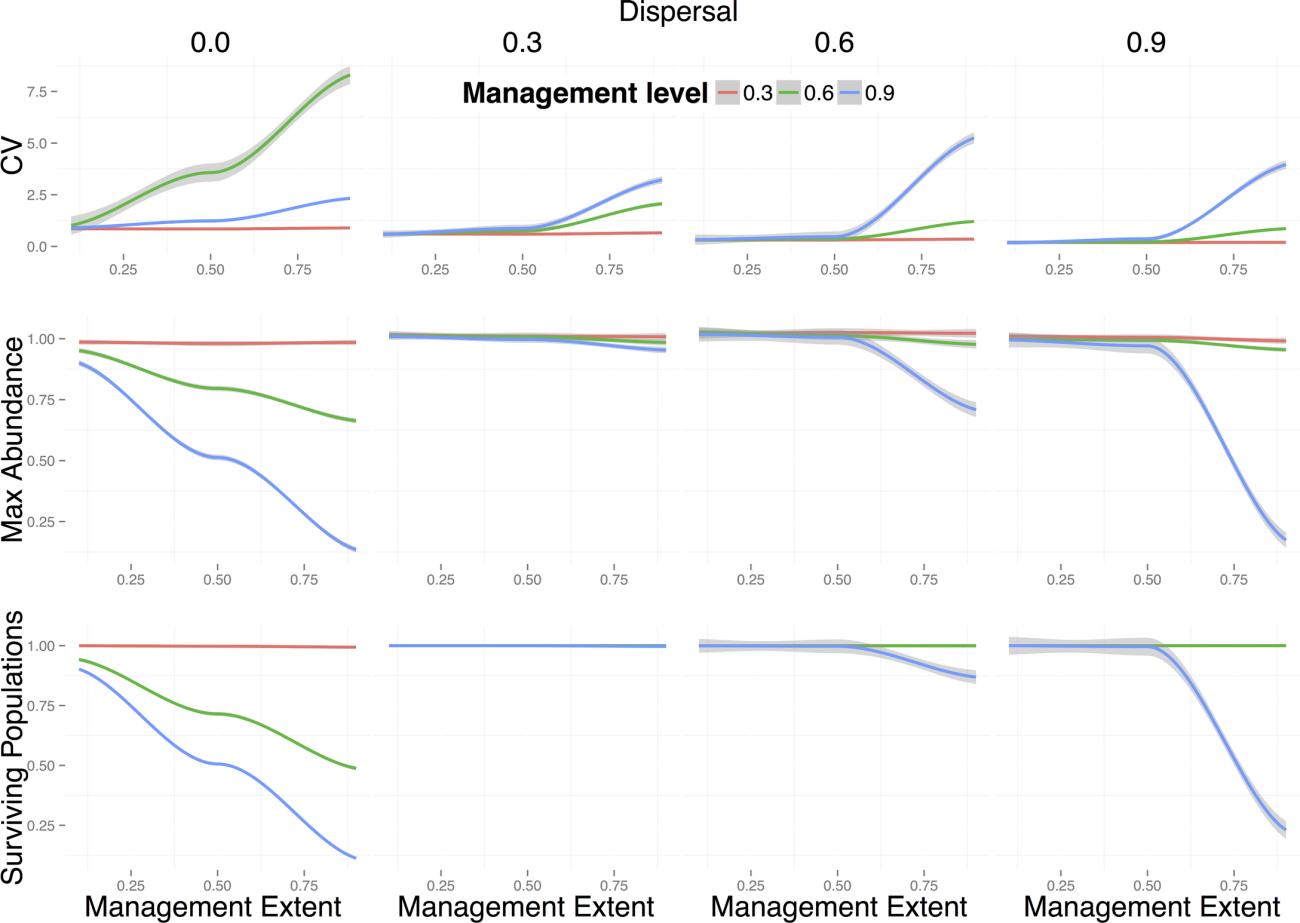
Effects of general population reduction on real-world rabbit metapopulations. Change in metapopulation stability (i.e. proportional change in coefficient of variation -CV-) (top row), change in maximum metapopulation abundance (middle row), and population persistence (i.e. fraction of surviving local populations) (bottom row) for different values of management extent (*e*), level (*l*), and different levels of dispersal (see methods). Lines are local polynomial regression fits to 100 replicates for each of the 10 sample landscapes studied and shadows around them represent the standard error of the mean. Colours represent different levels of spatial management (*l*): red = 0.3, green = 0.6, blue = 0.9. Management extent is the fraction of local populations that have been managed.

The effect of directly removing rabbits only became noticeable on the change in maximum metapopulation abundance for large management extents (*e* = 0.9, i.e., 90% of the local populations are managed) (Fig 4). The efficiency of population reduction was amplified for very low (i.e., no dispersal outside of a 5km^2^ grid cell) and very high dispersal rates (i.e. fraction of individuals dispersing from local populations) (*d* = 0.9). In the case of very high dispersal rates, this effect was only amplified when large levels of management were applied (i.e. when 90% of the targeted local populations is removed). This was probably due to the fact that when only a small fraction of the population remains in local communities, and a large fraction of the already small population disperses (*d* = 0.9), the chances of local populations going extinct increased due to demographic stochasticity. In contrast, intermediate levels of dispersal resulted in resilience of the metapopulation to this type of management. Management of populations via general population reduction had a noticeable effect on metapopulation stability, even for intermediate levels of dispersal, which did not affect maximum abundance or the fraction of surviving populations (Fig 4).

**Fig 5:**
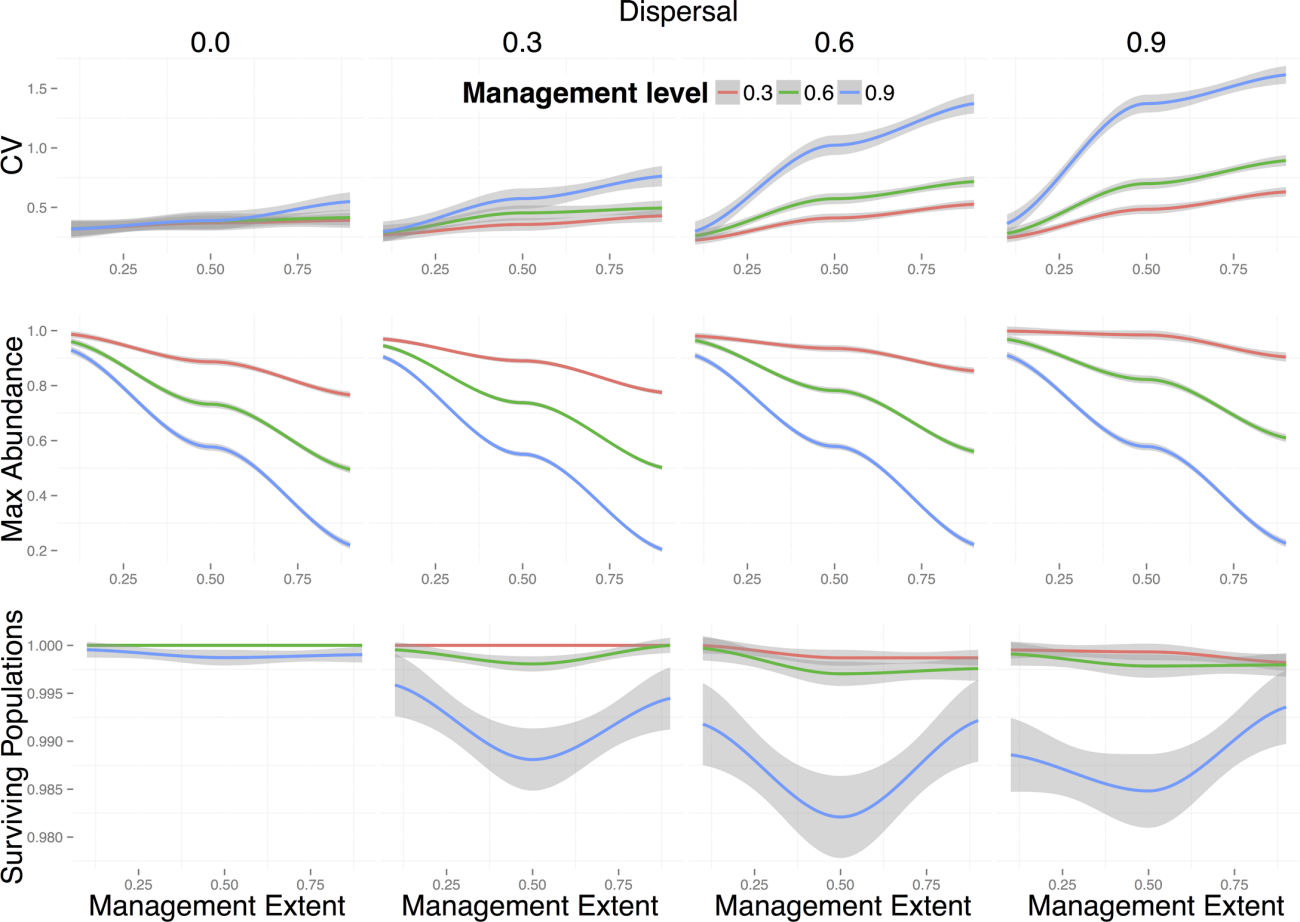
Effects of reduction of an obligate resource on real-world rabbit metapopulations. Change in metapopulation stability (i.e. proportional change in coefficient of variation -CV-) (top row), change in maximum metapopulation abundance (middle row), and population persistence (i.e. fraction of surviving local populations) (bottom row) for different values of management extent (*e*), level (*l*), and different levels of dispersal (see methods). Lines are local polynomial regression fits to 100 replicates for each of the 10 sample landscapes studied and shadows around them represent the standard error of the mean. Colours represent different levels of spatial management (*l*): red = 0.3, green = 0.6, blue = 0.9. Management extent is the fraction of local populations that have been managed.

Decrease in metapopulation abundances resulting from the reduction of obligate resources were most strongly influenced by management extent (*e*), even for small levels of management (*l*), with no differences among different dispersal levels (Fig 5, middle row). The outcome of this type of management was largely insensitive to dispersal because dispersing individuals were likely to encounter patches with low availability of their obligate resource.

In situations where the obligate resource was heavily reduced (*l* = 0.9, i.e. 90% reduction in *K*) we observed an inverted hump-shaped relationship between population persistence (i.e. the fraction of surviving populations) and extent of management for intermediate to high levels of dispersal (Fig 5, bottom row). Despite these differences being small (between 0.5% and 2% of difference in persistence), this result shows that management strategies applied over intermediate levels of spatial extents can be more effective at controlling rabbit metapopulations by altering the source-sink dynamics of the metapopulation. As expected, reductions of the obligate resource in real landscapes with unconnected populations, did not affect the metapopulation stability (first plot of upper row on Fig 5). This is because local populations are independent of each other when there is no dispersal. For intermediate levels of dispersal, metapopulation stability increased with management level (*l*) and extent (*e*), as shown by BRT partial effects plots (S4 Fig). Dispersal among local populations thus has an important effect on metapopulation stability.

The effects of different spatial management strategies (*random*, *correlated*, *hub*) on the change in maximum abundance of real-world rabbit metapopulations subjected to general population reductions were most noticeable for small metapopulations (e.g., rightmost plot in S5 Fig − rabbit metapopulation consisting of 11 local populations). There were nonetheless some noticeable effects for intermediate metapopulation sizes (e.g., middle plot in S5 Fig − metapopulation consisting of 662 local populations). Larger rabbit metapopulations were more resilient to different management strategies (S5 Fig) due to the highly homogeneous and largely connected topology of the network of populations. This is because the proximity of local populations increases when metapopulation connectivity is higher. The effect of initial metapopulation size on the effectiveness of different spatial management strategies is thus mediated by landscape connectivity, whereby different spatial management actions only affects population abundance if metapopulation connectivity is not too large.

The effects of reducing an obligate resource on metapopulation stability and persistence were noticeable from intermediate levels of dispersal and management onwards (e.g. *d* = 0.3 and *l* = 0.6) (Fig 5). As for the case of general population reduction, when reducing an obligate resource, different spatial management strategies yielded different outcomes only in metapopulations with a small number of local populations. Again this is because these populations were more sparsely distributed across the landscape, therefore making the metapopulation less connected (S6 Fig).

Collectively, these results based on real-world landscapes show that for both management actions (general population reduction and reduction of an obligate resource), regional (*correlated* and *hub*) and local (*random*) management strategies are likely to do equally well when managing invasive rabbit populations, except when rabbit metapopulation sizes are small.

## Discussion

Managing invasive species at landscape scales poses an important challenge to ecologists, conservation biologists and land managers. A key focus of landscape-scale management is on how to prioritise management efforts from local to regional scales in order to identify effective strategies for invasive species control; and in doing so, optimise the allocation of resources to management actions. Landscape-scale management strategies are commonly used to manage invasive species (e.g., [16–17]). However, it remains largely unknown how to best employ systematic spatial management designs that take into account the metapopulation structure of the established invasive species. By exploring interactions between different metapopulation structures and spatial management strategies, we found that spatial planning of management actions is most important for small metapopulation networks with heterogeneous distributions of links and low connectivity.

Heterogeneity in metapopulation structure and connectivity can greatly affect the outcome of management actions. As rabbit populations are typically homogeneous throughout the landscape for much of its invasive range in Australia, intensive spatial planning of management effort will not necessarily increase the efficiency of rabbit management. In situations where metapopulations are more heterogeneously connected, spatial management strategies that focus on more centrally located populations will yield the best management outcomes. This is because these more centrally located populations can provide an important source of immigrants to other populations. Our results highlight the importance of simulation and theoretical approaches to invasion biology.

Metapopulation theory has traditionally focused on the effect of spatial dispersal processes among local populations on regional species persistence [22]. We extend these findings to metapopulation stability by showing that low dispersal rates have the potential to destabilise regional populations when they are heavily managed (or perturbed) at the local scale. However, this effect is sensitive to the type of management (perturbation) being applied to the metapopulation. When a reduction in the availability of an obligate resource (which also enforces population reduction) is the source of the perturbation, the opposite trend was observed: more variation in population sizes and hence less metapopulation stability at high dispersal levels. These findings emphasize the importance of metapopulation connectivity for ecological stability [47], and point to the fact that the efficacy of different management strategies will vary in relation to the dispersal habits of the focal species.

The interaction between management action (population reduction vs. resource reduction), dispersal levels (*d)*, management extent (*e*) and level (*l*), spatial management strategy (*s*), and network topology produced complex responses in local and regional population dynamics, affecting the outcome of control measures in diverse ways. For example, when reducing local populations, only *star* or *scale-free* network topologies can be managed in an efficient way with low to intermediate levels of management. In contrast, for the other network configurations large levels of control (and therefore economical resources) are required for efficient management. Furthermore, those network topologies that are more amenable to management can cause population abundance to respond in a non-linear way to the strength of management intervention. We show that control efficiency increases rapidly when the management extent increases beyond an intermediate level. This is reminiscent of a critical threshold and suggests that populations might become disconnected when management extent is large. In the model presented here, however, metapopulation structure is conserved throughout the simulation, and no local population becomes isolated. This sharp increase in control efficiency is thus due to a weakening of source-sink metapopulation dynamics. When populations far from the core of the network are targeted, it is hard for them to receive immigrants from distant source populations (i.e., those with strong positive population growth). Once a sufficient amount of these are targeted, it becomes difficult for the whole metapopulation to recover from low densities. This observation emphasises the importance of management extent when controlling heterogeneously structured metapopulations of invasive species. Although it is well established that network structure can have large effects on dynamics [48], only recently have analyses similar to those shown here been used to study the management of contagious processes, such as diseases epidemiology [49–50]. Ours is a first attempt to use these ideas to understand how invasive species populations will react to local versus regional spatial perturbations and management strategies.

Our simulations suggest that reducing an obligate resource at the local population scale (modelled here as a reduction in carrying capacity) is a better strategy to keep rabbit numbers low than general population reduction. This might be due to the highly resilient nature of the rabbit populations, which in this study are modelled using a Ricker logistic function. This model for population growth promotes a strong and immediate rebound from population sizes below carrying capacity, replicating the rapid recovery of rabbit populations in Australia after control from only a small number of founding individuals; and the quick recovery of rabbit populations from disease outbreaks [37,51].

In south-western Western Australia (our study area), reducing an obligate resource using, for example warren ripping, is less feasible then in other regions of Australia. This is because rabbits tend not to nest in warrens in Western Australia. Control strategies that incorporate resource removal are not desirable since removing large amounts of shelter habitat (shrubland and open woodlands) is likely to result in detrimental effects on native communities. In these cases our model nonetheless identifies suitable courses of action, including maximising lethal control. Reduced access to resources has been shown as a potentially effective control measure to other highly invasive species in Australia, such as the cane toad [52]. Our framework provides a practical way of incorporating this information into landscape-level management strategies.

The effect of management strategy was weak in simulations using metapoulations of real-world landscapes from Western Australia compared to model metapopulation networks. This was probably because rabbit habitat in these landscapes tended to be more homogenous (at least at 5km x 5km grid cell spatial scale), with local populations tending to be clustered together in almost all the landscapes studied. Spatial scale might play a role on the shape of metapopulation structure. At much larger spatial extents than those considered here, a more heterogeneous structure is likely to emerge for rabbit metapopulations in Australia [21], whereby 5km x 5km grid cells are clustered together in large panmictic populations, and collections of those are connected via dispersal. Conversely, important inter-warren structure in local rabbit populations can occur at finer spatial scales than the ones used in this study.

Habitat occupation for rabbits in our models was approximated with high-to-moderate uncertainty due to the absence of more detailed survey data. Furthermore, these spatial estimates of occurrence needed to be aggregated at larger scales. Therefore it is possible that the real-world landscapes used are much more heterogeneous then those used in our analyses. These considerations might affect the outcome of our comparison of spatially-explicit management strategies and deserves further investigation. Our results based on model landscapes suggest that the spatial management strategy adopted will be greater in more heterogeneous landscapes, where the effects of source-sink dynamics can strongly affect metapopulation persistence. Our real-world metapopulations behave more like *ring*, *neighbours*, or *ring-random* model networks in response to perturbations because of homogeneous distributions of links among nodes.

Our model constitutes a simple approach for identifying the best possible management strategy/strategies for invasive species based on the structure connectivity between local populations and the amount of control capacity at hand. This information is important for devising efficient landscape-oriented management strategies for invasive species [15]. In Australia, it is common for invasive species managers to adopt several control measures at the same time, particularly for the management of wild rabbit populations [23]. Future work should target the effects of different control actions acting in tandem and how the interplay between these management actions and landscape-oriented management strategies affects the outcome of invasive species control. This will complement our study and help managers develop more coherent strategies for controlling invasive species. Furthermore, adding an economic component to this framework would improve its usefulness to managers. Data exists on the cost of control methods for rabbits in Australia, including baiting (causing population reduction) and warren ripping (causing a reduction in carrying capacity) [53–54]. This information could be directly used in future applications of our model to test the economic feasibility of different management scenarios and identify potential trade-offs between management efficacy and cost. Despite these limitations, our new approach provides a framework for better understanding the interplay between landscape topology and management strategies for invasive species management. In doing so, it adds to other platforms available for this type of study (reviewed in [55]), and could be very easily extended to account for the influence of human-induced climate change on invasiveness [56]. Our framework can also be applied to conservation of wildlife populations and to the analysis of the effects of human induced perturbations on natural populations [11–12].

Rabbits are present throughout much of Australia, and in many areas they tend to occur homogenously across the landscape [21]. Therefore, our observations for Western Australia are useful beyond the study region. Furthermore, in areas where rabbit populations tend to be more fragmented, such as at their northern Australian range boundary (latitude = ~ -18 to -16) we would expect that choice of management strategy would have a much larger influence on effective rabbit control. In such situations (where populations are more fragmented), where metapopulation structure yields heterogeneous distributions of the links amongst local populations, management strategies targeting local populations will be most efficient.

## Conclusion

The connection of local species populations into metapopulation networks means that population recovery from management actions or natural perturbations alike can be compensated by both population growth and dispersal. Here we have shown that efforts to manage invasive species can be improved by accounting for the interaction between metapopulation network topology and spatial management strategies, along with dispersal behaviour of the target species. In some landscape configurations, regional management strategies are more efficient than local (more random) ones, whereas the opposite is never the case. This suggests that landscape-oriented management strategies should account for the network structure of local populations to design more efficient regional management optima. These results also have consequences for conservation biology and more specifically conserving endangered species by maintaining strategically located local populations that are important for promoting regional persistence.

## Supporting Information

S1 Appendix Source code for the software implementation of the metapopulation model proposed. The archive includes source code, readme file explaining how to run it, and the input files necessary to run the simulations.
(ZIP)

S1 Fig Effects of general population reduction on model metapopulations with low dispersal and intermediate management level. Relative change in maximum abundance is plotted against management extent (*e*) for different values of spatial management strategy (*s*). Dispersal (*d*) and management level (*l*) are constant (*d* = 0.1 and *l* = 0.6). Lines are a local polynomial regression fit to 100 replicates of each value of management extent for each s. Shadows around them are standard error of the mean.
(PDF)

S2 Fig Effects of general population reduction on model metapopulations with high dispersal and management level. Change in maximum abundance is plotted against management extent (*e*) for different values of spatial management strategy (*s*). Dispersal (*d*) and management level (*l*) are constant (*d* = 0.6 and *l* = 0.9). Lines are a local polynomial regression fit to 100 replicates of each value of management extent for each s. Shadows around them are standard error of the mean.
(PDF)

S3 Fig Relative influence of predictor variables describing management scenarios on the fraction of surviving populations. Plots in A and B show the relative importance of the explanatory variables dispersal (*d*), management extent (*e*), management level (*l*), and spatial management strategy (*s*), for the two management actions studied: general population reduction, and reduction in resource availability, respectively. Numbers in x-axis represent the percentage of influence.
(PDF)

S4 Fig Relative influence of predictor variables describing management scenarios on coefficient of variation of population-scale abundances. Plots in A and B show the relative importance of the explanatory variables dispersal (*d*), management extent (*e*), management level (*l*), and spatial management strategy (*s*), for the management actions studied: general population reduction, and reduction in resource availability, respectively. Numbers in x-axis represent the percentage of influence.
(PDF)

S5 Fig Effects of general population reduction of rabbits on real metapopulations for different spatial management strategies. Change in the maximum population abundance is plotted against the spatial extent of management (*e*) for different spatial management strategies (*s*), with dispersal (*d*) = 0.6 and management level (*l*) = 0.9. Lines are a local polynomial regression fit to 100 replicates of each value of management extent for each s. Shadows around them are standard error of the mean. Number of local populations is 1619, 662 and 11 for the populations corresponding to the plots on the left, centre, and right respectively. Original landscapes (1, 4, and 8) shown in Fig 2 in the main text. Colours represent different spatial management strategies (*s*): red = random, green = correlated, blue = hub.
(PDF)

S6 Fig Effects of decrease in local carrying capacity on real-world metapopulations for different spatial management strategies. Change in the maximum population abundance is plotted against the spatial extent of management (*e*) for different spatial management strategies (*s*), with dispersal (*d*) = 0.3 and management level (*l*) = 0.6. Lines are a local polynomial regression fit to 100 replicates of each value of management extent for each spatial management strategy. Shadows around them are standard error of the mean. Number of local populations is 1619, 662 and 11 for the populations corresponding to the plots on the right, centre, and left respectively. Original landscapes (1, 4, and 8) shown in Fig 2 in the main text.
(PDF)

S1 Table Turretfield (South Australia) sample site time series of rabbit population abundances. Parameters values for the demographic model used in this study were estimated from this time series (see main text).
(CSV)
