# Supplementary figures and images for: A Landscape Approach to Invasive Species Management
Source: PLoS One. 2016 Jul 29;11(7):e0160417. doi: 10.1371/journal.pone.0160417 (PMC4966913; doi:10.1371/journal.pone.0160417)

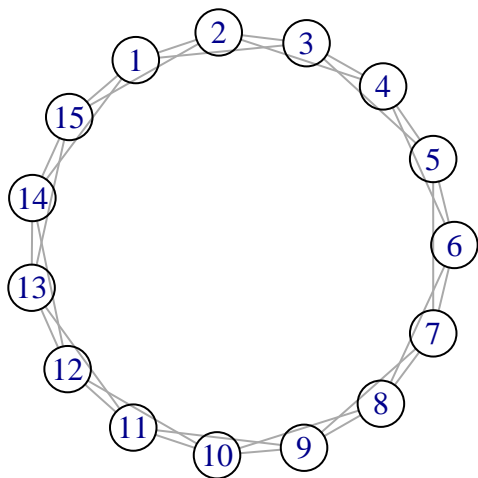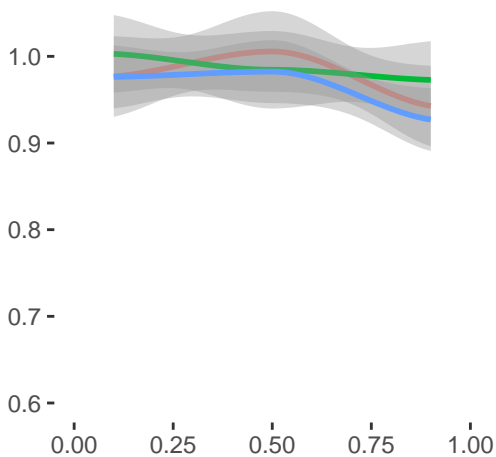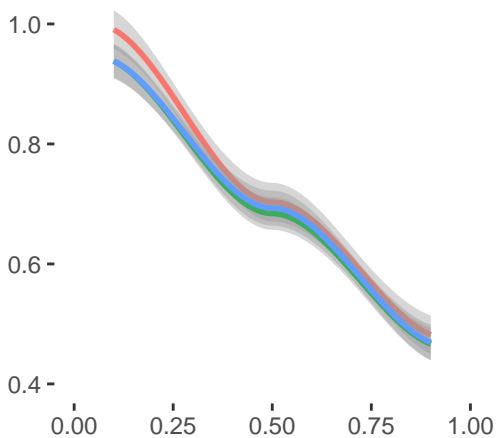

Management Extent

Supplement: S1 Appendix — The archive includes source code, readme file explaining how to run it, and the input files necessary to run the simulations. (ZIP) [file pone.0160417.s001.zip › appendix/neighbours-test.pdf]

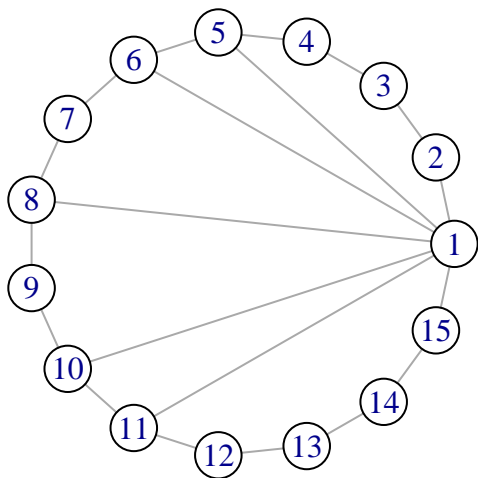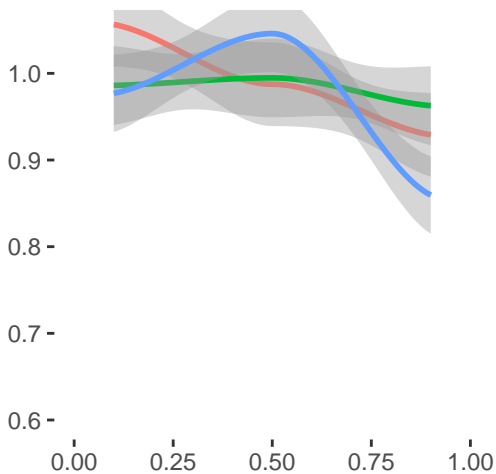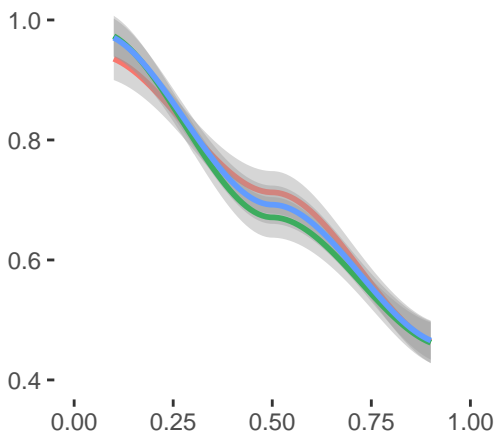

Management Extent

Supplement: S1 Appendix — The archive includes source code, readme file explaining how to run it, and the input files necessary to run the simulations. (ZIP) [file pone.0160417.s001.zip › appendix/ring-hub-test.pdf]

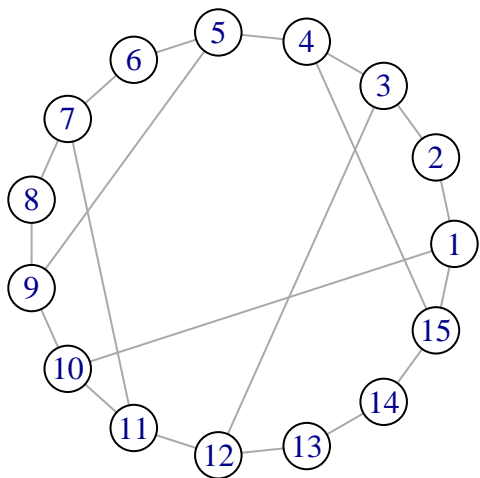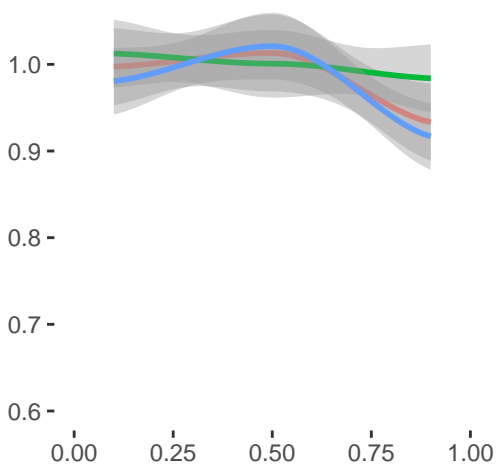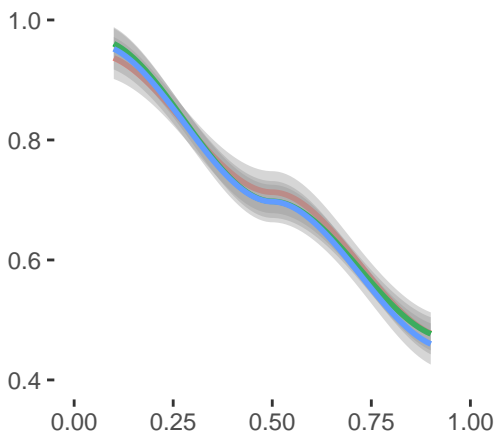

Management Extent

Supplement: S1 Appendix — The archive includes source code, readme file explaining how to run it, and the input files necessary to run the simulations. (ZIP) [file pone.0160417.s001.zip › appendix/ring-random-test.pdf]

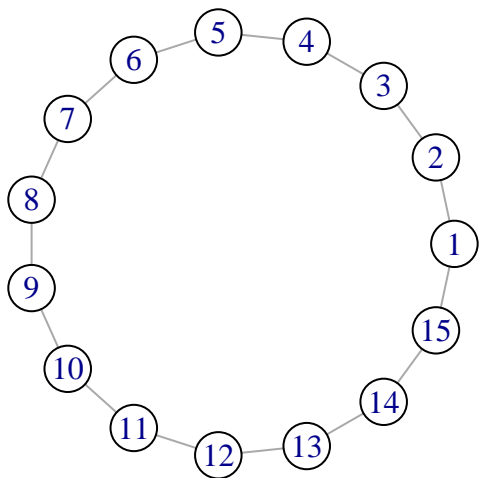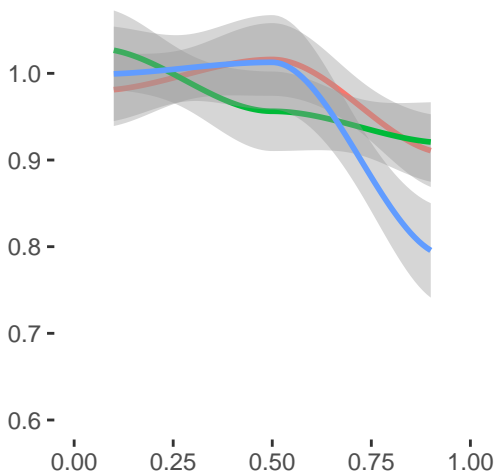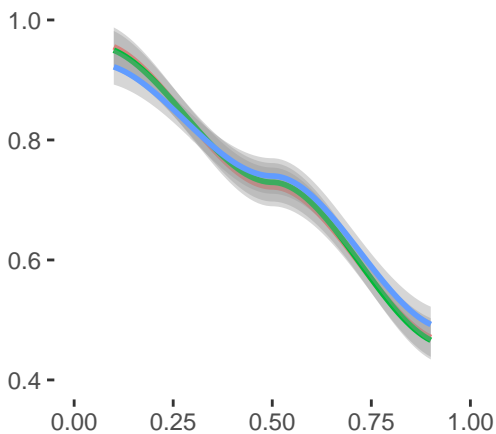

Management Extent

Supplement: S1 Appendix — The archive includes source code, readme file explaining how to run it, and the input files necessary to run the simulations. (ZIP) [file pone.0160417.s001.zip › appendix/ring-test.pdf]

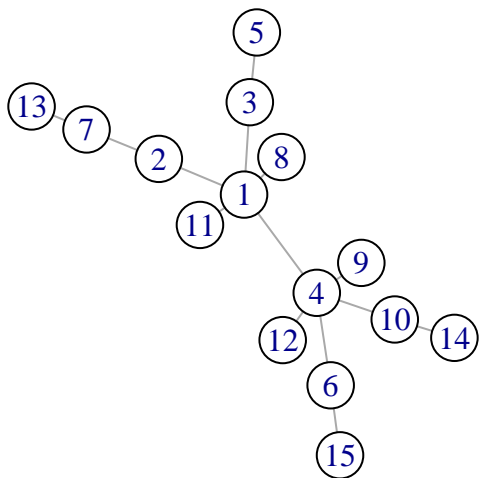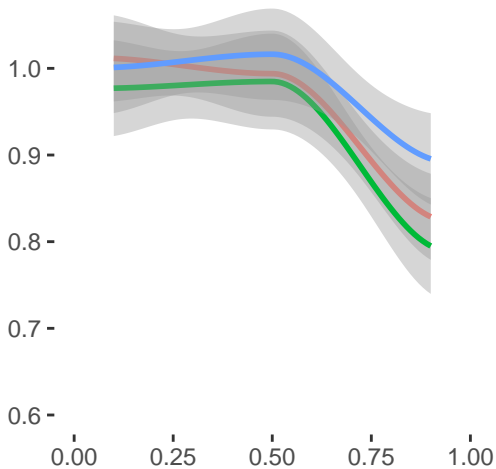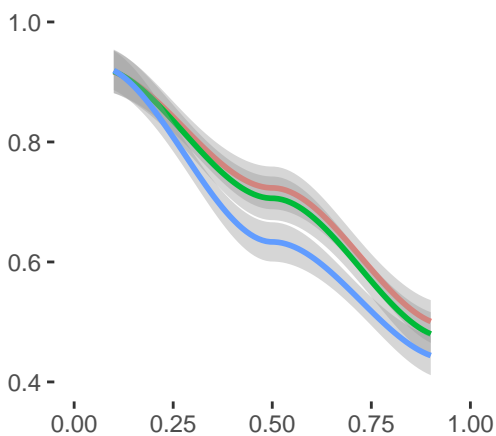

Management Extent

Supplement: S1 Appendix — The archive includes source code, readme file explaining how to run it, and the input files necessary to run the simulations. (ZIP) [file pone.0160417.s001.zip › appendix/scale-free-test.pdf]

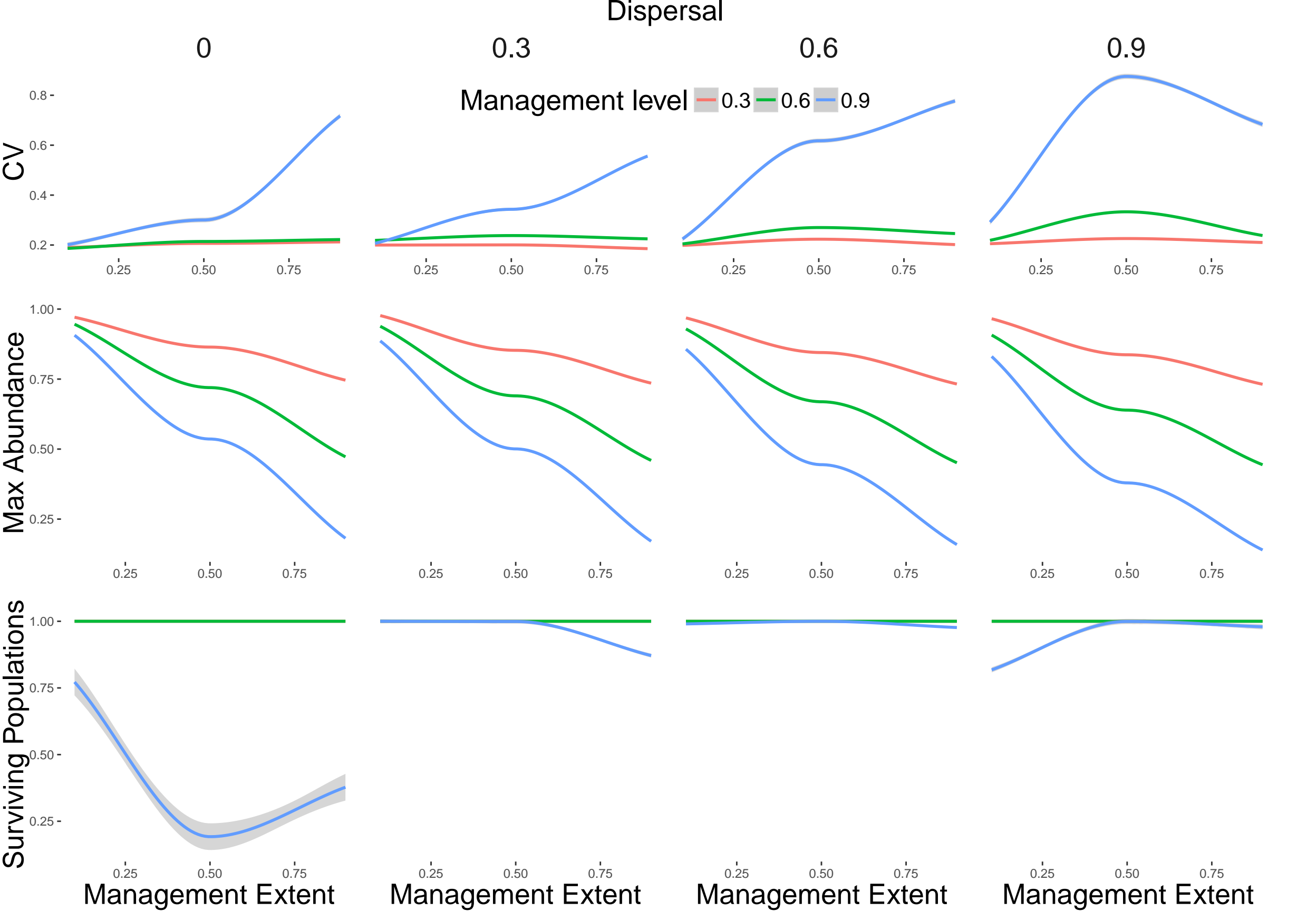

Supplement: S1 Appendix — The archive includes source code, readme file explaining how to run it, and the input files necessary to run the simulations. (ZIP) [file pone.0160417.s001.zip › appendix/test.pdf]

# Management Strategy

random correlated hub

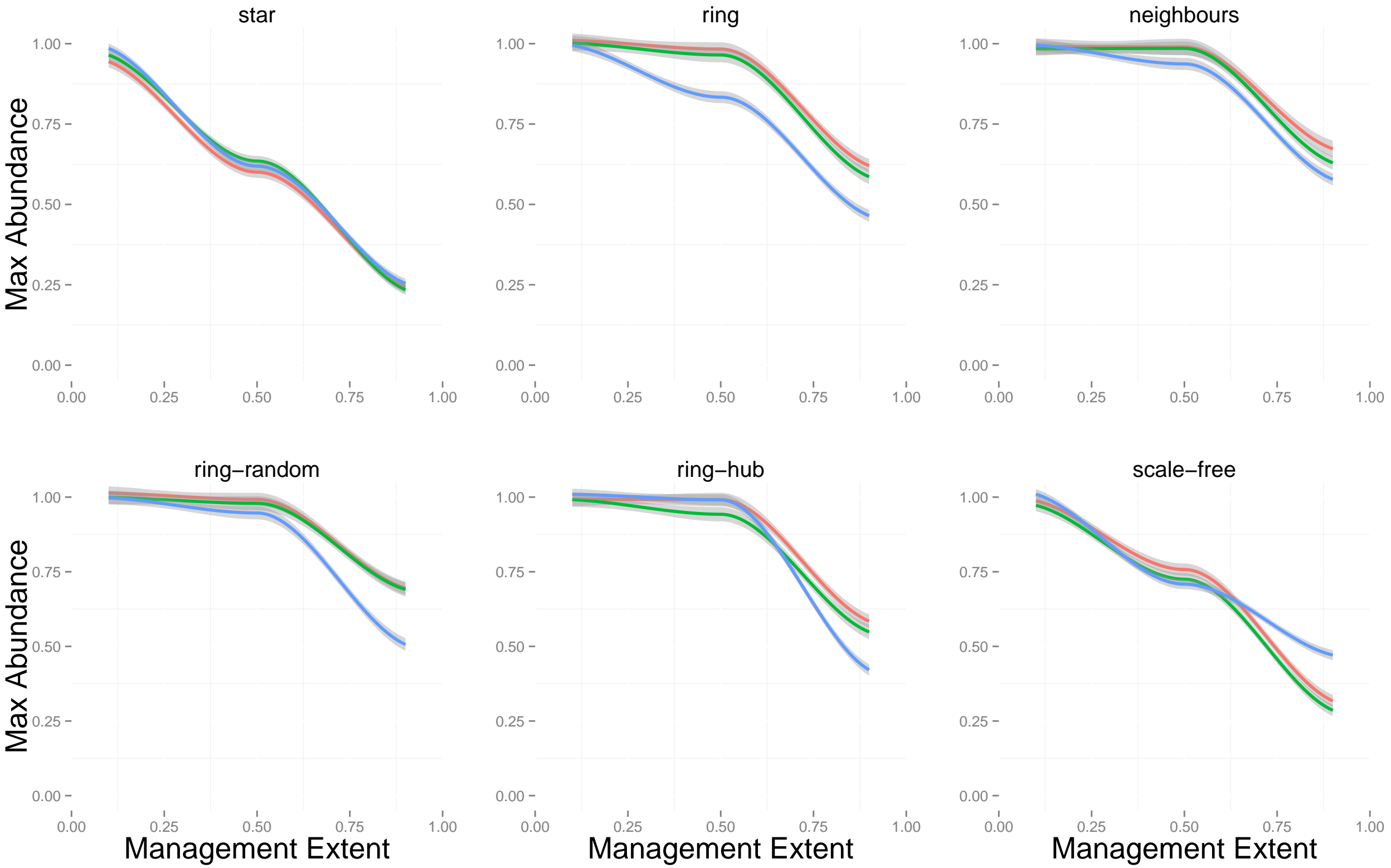

Supplement: S1 Fig — Relative change in maximum abundance is plotted against management extent (e) for different values of spatial management strategy (s). Dispersal (d) and management level (l) are constant (d = 0.1 and l = 0.6). Lines are a local polynomial regression fit to 100 replicates of each value of management extent for each s. Shadows around them are standard error of the mean. (PDF) [file pone.0160417.s002.pdf]

# Management Strategy

random correlated hub

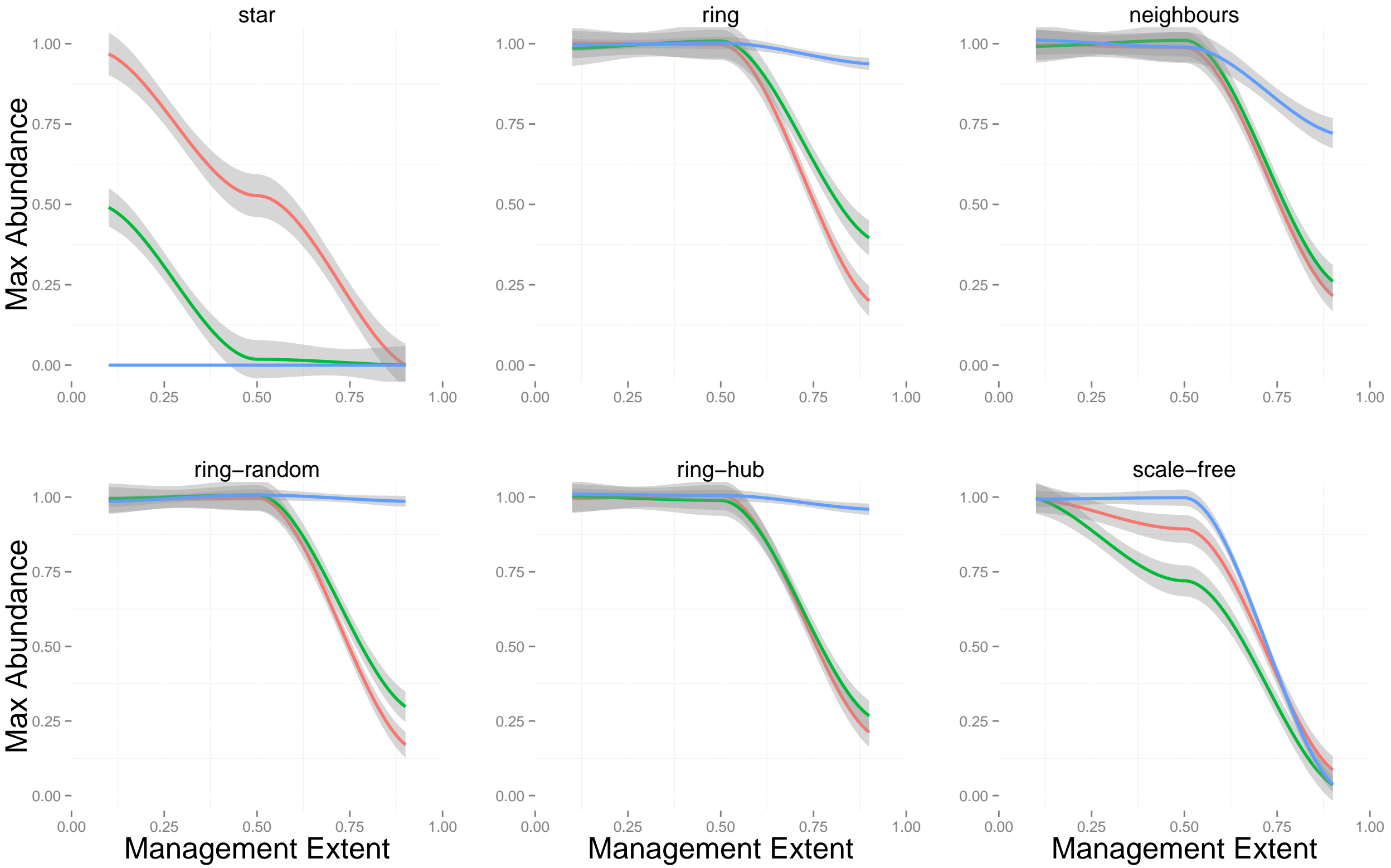

Supplement: S2 Fig — Change in maximum abundance is plotted against management extent (e) for different values of spatial management strategy (s). Dispersal (d) and management level (l) are constant (d = 0.6 and l = 0.9). Lines are a local polynomial regression fit to 100 replicates of each value of management extent for each s. Shadows around them are standard error of the mean. (PDF) [file pone.0160417.s003.pdf]

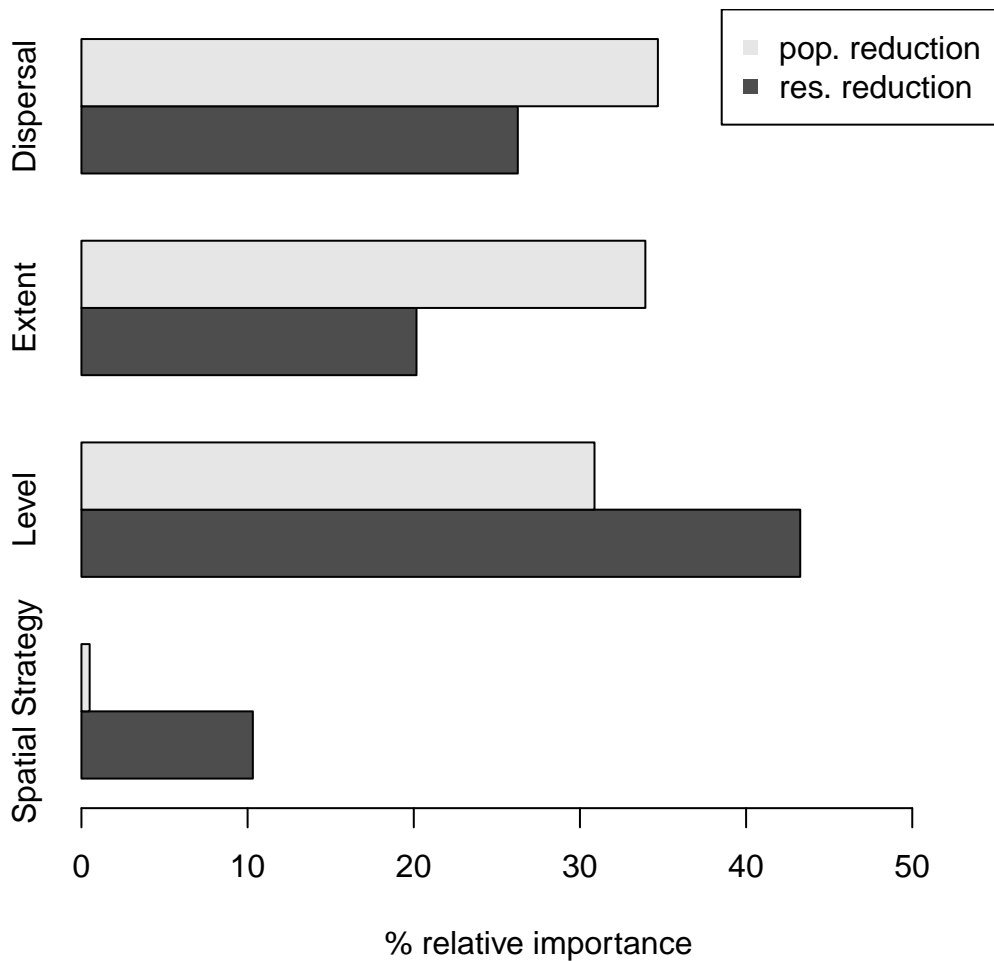

Supplement: S3 Fig — Plots in A and B show the relative importance of the explanatory variables dispersal (d), management extent (e), management level (l), and spatial management strategy (s), for the two management actions studied: general population reduction, and reduction in resource availability, respectively. Numbers in x-axis represent the percentage of influence. (PDF) [file pone.0160417.s004.pdf]

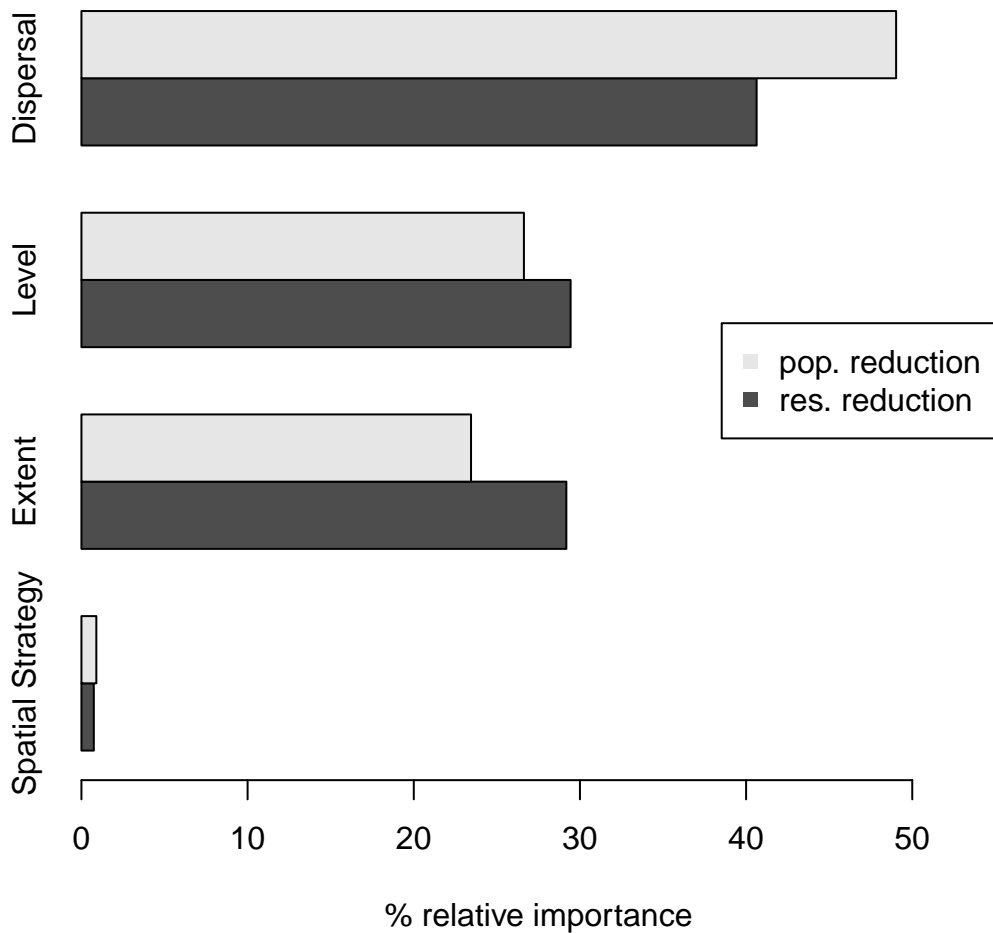

Supplement: S4 Fig — Plots in A and B show the relative importance of the explanatory variables dispersal (d), management extent (e), management level (l), and spatial management strategy (s), for the management actions studied: general population reduction, and reduction in resource availability, respectively. Numbers in x-axis represent the percentage of influence. (PDF) [file pone.0160417.s005.pdf]

# Management Strategy

random correlated hub

Max Abundance

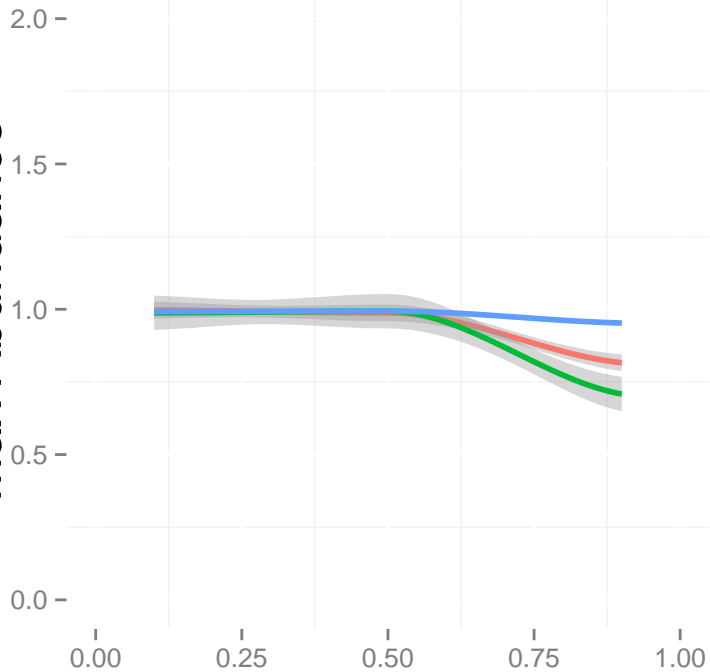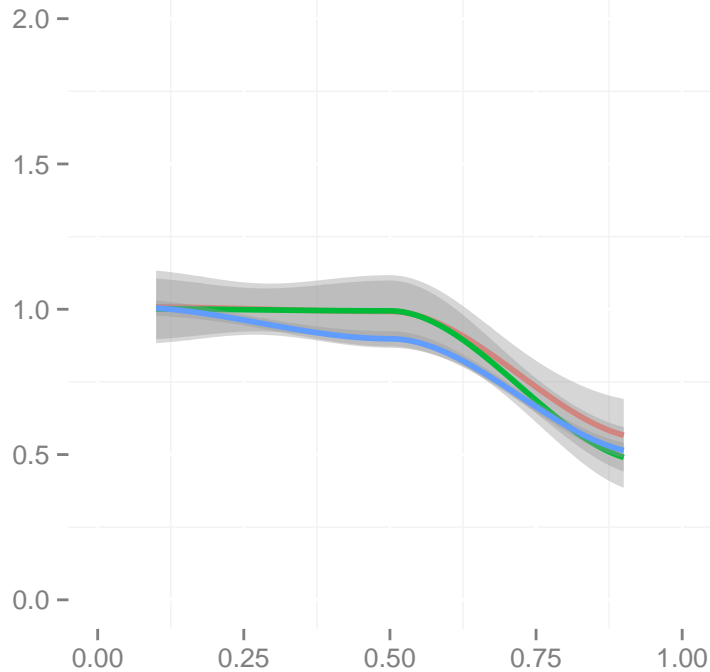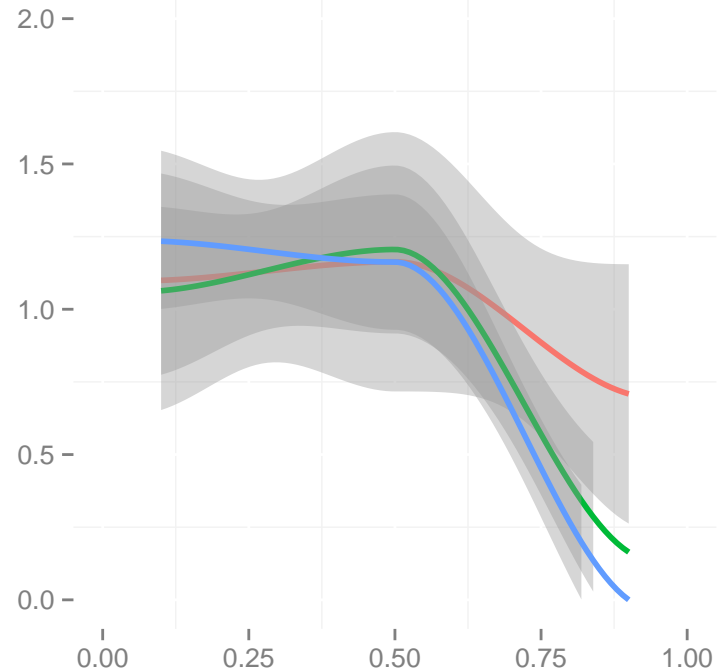

Supplement: S5 Fig — Change in the maximum population abundance is plotted against the spatial extent of management (e) for different spatial management strategies (s), with dispersal (d) = 0.6 and management level (l) = 0.9. Lines are a local polynomial regression fit to 100 replicates of each value of management extent for each s. Shadows around them are standard error of the mean. Number of local populations is 1619, 662 and 11 for the populations corresponding to the plots on the left, centre, and right respectively. Original landscapes (1, 4, and 8) shown in Fig 2 in the main text. Colours represent different spatial management strategies (s): red = random, green = correlated, blue = hub. (PDF) [file pone.0160417.s006.pdf]

**Management Strategy** random correlated hub

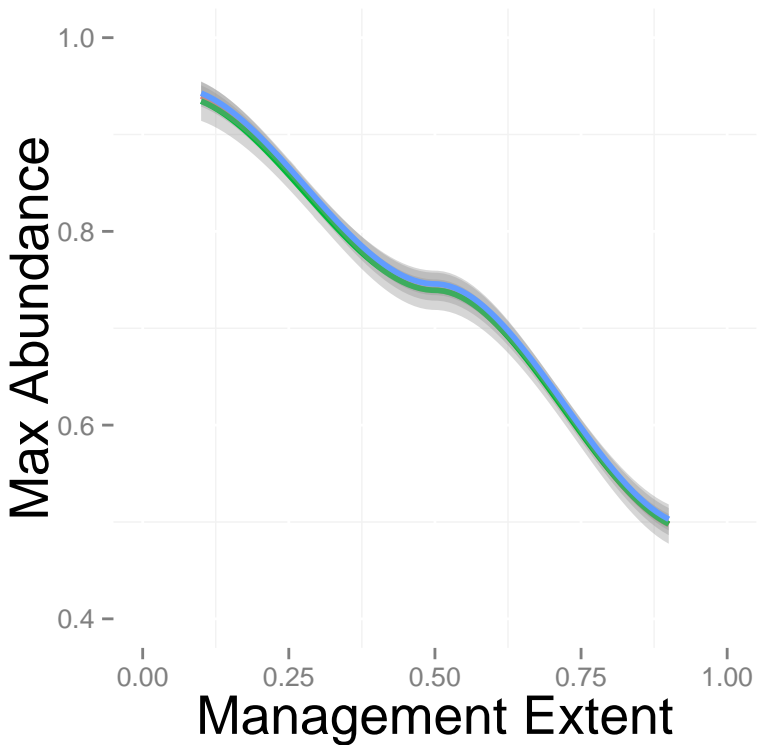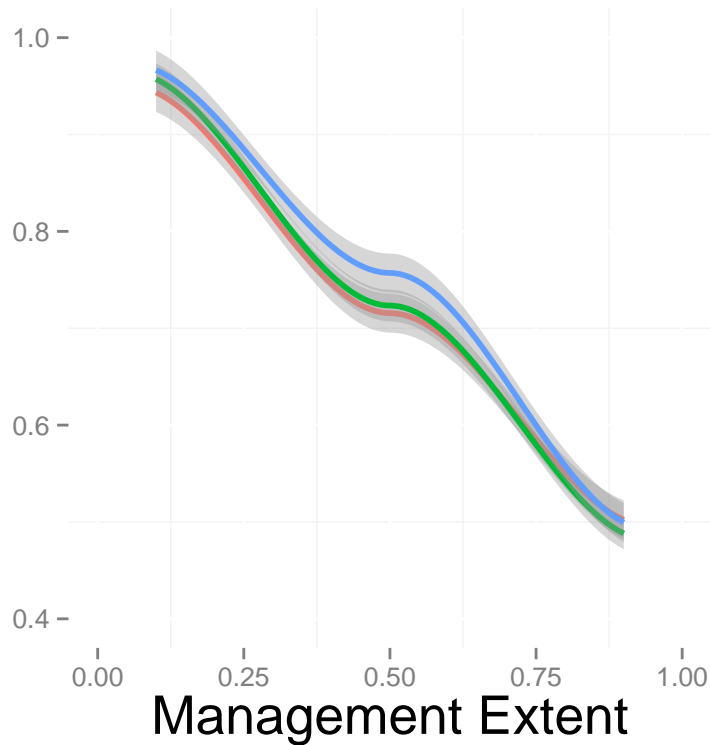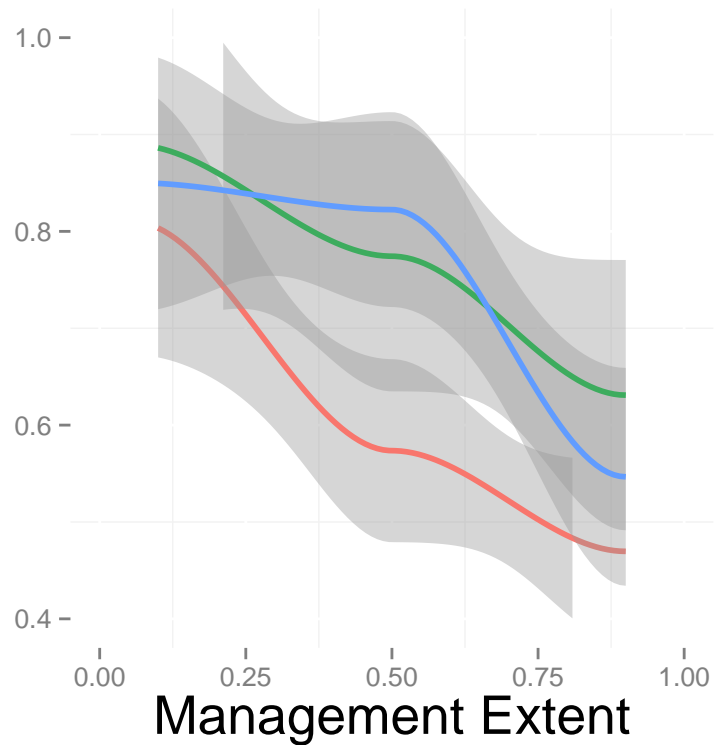

Supplement: S6 Fig — Change in the maximum population abundance is plotted against the spatial extent of management (e) for different spatial management strategies (s), with dispersal (d) = 0.3 and management level (l) = 0.6. Lines are a local polynomial regression fit to 100 replicates of each value of management extent for each spatial management strategy. Shadows around them are standard error of the mean. Number of local populations is 1619, 662 and 11 for the populations corresponding to the plots on the right, centre, and left respectively. Original landscapes (1, 4, and 8) shown in Fig 2 in the main text. (PDF) [file pone.0160417.s007.pdf]
